# Acute Myeloid Leukemia With *NPM1* Mutation Presenting With Rapidly Progressing Hypereosinophilia

**DOI:** 10.1155/crh/5125740

**Published:** 2025-06-18

**Authors:** S. Einarsdottir, G. Orrsjö, L. von Bahr, A. Staffas, L. Fogelstrand

**Affiliations:** ^1^Department of Hematology and Coagulation, Region Västra Götaland, Sahlgrenska University Hospital, Gothenburg, Sweden; ^2^Institute of Medicine, University of Gothenburg, Sahlgrenska Academy, Gothenburg, Sweden; ^3^Department of Laboratory Medicine, Institute of Biomedicine, University of Gothenburg, Sahlgrenska Academy, Gothenburg, Sweden; ^4^Department of Clinical Genetics and Genomics, Region Västra Götaland, Sahlgrenska University Hospital, Gothenburg, Sweden; ^5^Department of Clinical Chemistry, Region Västra Götaland, Sahlgrenska University Hospital, Gothenburg, Sweden

## Abstract

Hypereosinophilia presents a significant clinical challenge. We describe a case of severe, rapidly progressing hypereosinophilia, with the white blood cell count increasing from 40,000/μL to over 130,000/μL within days, and 70% eosinophils on differential count. The patient initially presented with diffuse symptoms but developed eosinophilic myocarditis during hospitalization. Targeted next-generation sequencing identified a mutation in *NPM1* and according to the WHO 5^th^ edition criteria, the patient was diagnosed with acute myeloid leukemia (AML) with *NPM1* mutation. Whole genome and transcriptome sequencing revealed a concurrent fusion *ETV6*::*ACSL6*. This fusion has been previously described in myeloid diseases with eosinophilia. Despite initial deep response to AML treatment, reaching MRD-negativity for *NPM1*, the patient relapsed shortly after stem cell transplantation and died.

## 1. Introduction

Hypereosinophilia (> 1.5 × 10^9^/μL) can be seen in a wide range of diseases and can lead to severe organ damage due to eosinophilic tissue infiltration and the release of granules [1]. Most eosinophilia is reactive, caused by an overproduction of eosinophilopoietic cytokines such as interleukin 3 (IL-3), interleukin 5 (IL-5), or granulocyte-macrophage colony stimulating-factor (GM-CSF) [2]. Reactive eosinophilia can occur secondary to infections, atopic conditions, autoimmune disorders, medication, and malignancies [3, 4]. Primary hypereosinophilia, in contrast, results from clonal eosinophil proliferation and is associated with both myeloid and lymphoid neoplasms. Some well-defined entities include myeloid/lymphoid neoplasms with *PDGFRA*, *PDGFRB*, or *FGFR1* rearrangements, as well as *PCM1::JAK2* fusions, all of which involve tyrosine kinase activation [5].

Eosinophilia associated with nonspecific clonal cytogenetic or molecular abnormalities and increased bone marrow blasts (5%–20%) is classified as chronic eosinophilic leukemia not otherwise specified (CEL-NOS) [6]. If clonal proliferation is absent and secondary causes are ruled out, a diagnosis of idiopathic hypereosinophilic syndrome (HES) is often made.

## 2. Case Presentation

A 53-year-old woman sought medical attention due to fatigue, night sweats, and low-grade fever. Upon presentation to the regional hospital, a complete blood count (CBC) revealed a white blood cell (WBC) count of 44,000/μL, consisting predominantly of eosinophils (55%) and blast cells (7%), along with anemia (Hb 8.0 g/dL) and thrombocytopenia (33,000/μL). She had no significant medical history and was not on any medications. Clinical examination was unremarkable. Computed tomography (CT) scans of the thorax and abdomen were normal. A comprehensive infectious workup, including repeated fecal examinations for parasitic infections, was negative. In addition, ANA and ANCA screens were negative, and serum tryptase levels were within normal limits. No *JAK2*V617F or *BCR*::*ABL1* could be detected in peripheral blood.

**Figure 1:**
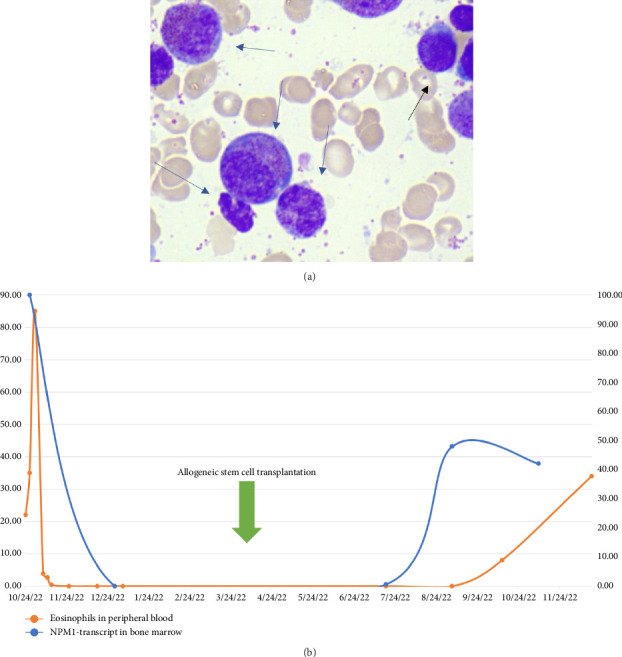
(a) Bone marrow aspirate showing a hypercellular marrow with prominent eosinophilia and numerous eosinophilic precursors. An increased number of myeloblasts is also observed. The black arrow indicates a blast cell, and the gray arrow highlights eosinophils at various stages of maturation. (b) Correlation between peripheral blood eosinophil count and NPM1 transcript levels in bone marrow.

Bone marrow evaluation revealed a striking presence of eosinophils and eosinophilic precursors with 71% of the granulocytes being eosinophils and 6% myeloblasts (Figure 1(a)). Multiparameter flow cytometry confirmed the presence of 4% cells with atypical myeloid immunophenotype. The cells were weakly positive for CD45 and expressed CD34 (heterogeneously), CD117, HLA-DR, CD7, CD13, CD33^dim^, and CD71^dim^ but not CD10, CD11b, CD19, CD56, or the monocytic markers CD14, CD35, CD36, CD64, or IREM2. The partial CD34 expression was suggestive of some immunophenotypic diversity, but there were no distinct subpopulations. Remaining cells showed mature myeloid expression patterns, consistent with the majority being eosinophils.

Based on the clinical presentation, CEL was suspected. Shortly after admission, the patient developed chest discomfort and was found to have an abnormal electrocardiogram (ECG). Troponin I (TnI) levels rose progressively from 4700 to 5300 ng/L and then to 7700 ng/L, while NT-proBNP was elevated at 11,000 pg/mL. A transthoracic echocardiogram revealed discrete apical hypokinesia, prompting further evaluation. Cardiac magnetic resonance imaging (MRI) showed diffuse subepicardial edema and late gadolinium enhancement, consistent with eosinophilic myocarditis.

Over the next three days, the white blood cell count surged to 132,000/μL. While awaiting results for PDGFR rearrangements, empirical treatment was initiated, including prednisolone (1 mg/kg), hydroxyurea (1 g twice daily), and imatinib (400 mg once daily). The patient was subsequently transferred to our tertiary referral center for further management. Upon transfer, pulsed cytarabine (500 mg/m^2^ IV, total of six doses) was added to the regimen, and prednisone was switched to intravenous methylprednisolone (500 mg once daily). FISH showed no rearrangements of PDGFRB, FGRFR1, or PCM1::JAK2, and digital PCR for FIP1L1::PDGRFA was negative. G-banding revealed a complex karyotype including a translocation *t* (5; 12)(q31; p11.2) but no evidence for separate clonal populations.

Targeted next generation sequencing revealed three mutations with similar variant allele frequencies (VAFs of 38%–39%): a *DNMT3A* R882C mutation, an *NPM1* type A mutation, and a *CEBPA* frameshift mutation located just upstream of the first transactivation domain (TAD1) (*CEBPA*:c.68del p.(Pro23ArgfsTer137)). PCR with fragment analysis did not show any *FLT3*-ITD mutation.

Later, whole genome sequencing revealed multiple structural aberrations, including *t* (5; 12)(q31.1; p13.2); *ETV6*::*ACSL6*, and confirmed the previously detected mutations. As *NPM1* mutation is an AML-defining mutation according to the WHO 5^th^ edition [7], a diagnosis of AML was made.

Due to the cardiotoxicity of anthracyclines and the patient's recent eosinophilic myocarditis, standard daunorubicin–cytarabine induction was omitted.

Instead, therapy was initiated with two cycles of amsacrine (150 mg/m^2^ IV once daily for five days), cytarabine (100 mg/m^2^ IV once daily for five days), and etoposide (110 mg/m^2^ IV once daily for five days) (ACE regimen). Given the patient's history of palpitations and frequent premature ventricular contractions (PVCs) on ECG, amsacrine infusions were administered under continuous cardiac monitoring in the cardiology department. Complete hematological remission was achieved after the first ACE cycle, as confirmed by a bone marrow assessment, although measurable residual disease (MRD) was not evaluated at that time. After the second ACE cycle, MRD negativity was confirmed. Consolidation therapy was administered with one cycle of azacitidine (200 mg subcutaneously once daily for five days) and venetoclax (400 mg once daily for 14 days following an initial dose ramp-up).

The patient subsequently underwent allogeneic hematopoietic stem cell transplantation (allo-HSCT) with peripheral blood stem cells from a fully matched (10/10) unrelated donor. Conditioning was given with fludarabine and treosulfan 42 g/m^2^, GvHD-prophylaxis with antithymocyte globulin 4 mg/kg, and four doses of methotrexate and cyclosporine. The transplant course was uncomplicated, and the patient was discharged on day +29 with normalized peripheral blood counts. Cyclosporine was tapered over 4 months, and no acute or chronic GVHD developed.

Five months post transplantation, the patient had a molecular relapse based on *NPM1* RT-qPCR, with subsequent increased eosinophils in peripheral blood (Figure 1(b)). This was followed by a full leukemia relapse in the bone marrow within weeks. At the time of relapse, immunophenotyping revealed a similar expression pattern at diagnosis, with no clear signs of phenotypic evolution. G-banding also revealed the same complex karyotype including the *t* (5; 12)(q31; p11.2) translocation as detected at diagnosis without signs of clonal evolution.

The patient was treated with azacytidine + venetoclax and cytarabine following relapse, but the disease progressed rapidly, and she was transitioned to palliative care. Due to the aggressive course, donor lymphocyte infusion (DLI) could not be administered, and the patient died shortly thereafter.

**Figure 2:**
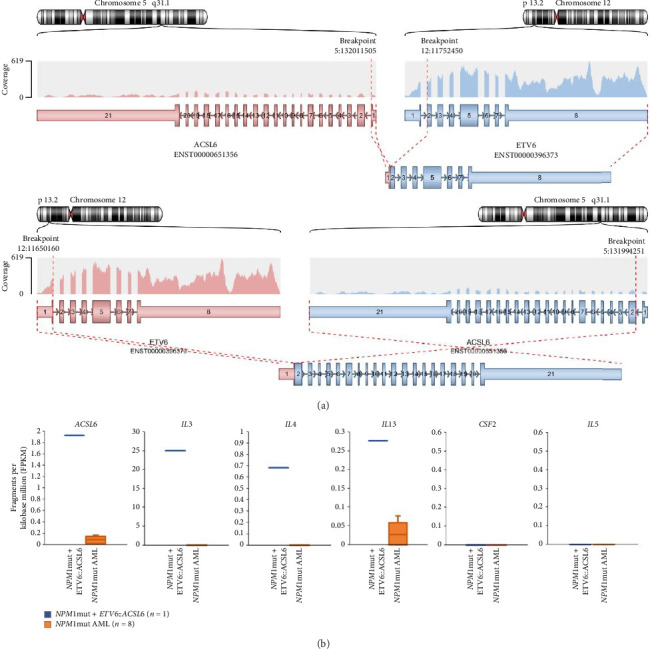
*ETV6*::*ACSL6*- and *ACSL6*::*ETV6*-fusion transcripts (a) and expression level of cytokine genes located in proximity to the fusion breakpoint on chromosome 5 (5p31.1) (b), both as detected by RNA sequencing.

To assess the effect of the *ETV6*::*ACSL6* fusion, whole transcriptome sequencing was performed. The *ETV6*::*ACSL6* fusion transcript along with a reciprocal *ACSL6*::*ETV6* transcript was detected, however, both out of frame (Figure 2). Expression of genes in the proximity of ACSL6 was compared with eight other AML with mutated *NPM1* from our institution. This analysis showed upregulation of *ACSL6* along with *IL3*, *IL4*, and *IL13* but not C2F2 or IL5.

## 3. Discussion

We report a case of acute myeloid leukemia (AML) with mutated *NPM1*, presenting with severe hypereosinophilia, organ involvement, and a low blast count. The aggressive clinical course was evident from the rapid white blood cell expansion and organ involvement, despite a modest increase in bone marrow blasts. Due to the atypical presentation, the initial genetic workup was not performed according to standard acute leukemia protocols. Consequently, the diagnosis of AML was only established weeks later, following the identification of an NPM1 mutation.

RNA sequencing identified two fusion transcripts: *ETV6::ACSL6* and *ACSL6::ETV6.* The *ETV6::ACSL6* fusion has been previously reported in myeloid neoplasms associated with hypereosinophilia [8–10]. In a review of cases with *ETV6*::*ACSL6*, a majority (10/12) had eosinophilia upon presentation [9]. However, only some reported cases have demonstrated a fusion transcript. In our case, both transcripts were out of frame, suggesting that they could not produce a functional protein. *ACSL6* encodes acyl-CoA synthetase long-chain family member 6, an enzyme involved in the conversion of fatty acids to acyl-CoA. The *ACSL6* locus is located near the cytokine gene cluster that includes GM-CSF, IL-3, and IL-5 [10], and the rearrangement might cause dysregulation of these mediators of eosinophilic proliferation. Indeed, we could see an upregulation of the genes encoding for IL-3, IL-4 and IL-13, all cytokines produced by eosinophils, compared with cases of AML with mutated *NPM1* without any sign of *t* (5; 12). This finding supports a hypothesis of dysregulation of genes important for eosinophils. However, a limitation of our analysis is that cytokine levels in serum were not available, and we could, therefore, not confirm whether transcriptional changes were reflected at the protein level. In acute leukemia, *ETV6* is frequently involved in fusion with other genes, and for some fusions, the mechanism for leukemia is not the production of a functional fusion protein but rather affected gene expression, as we have previously shown for AML with *t* (7; 12)(q36; p13), resulting in the rearrangement of *ETV6* and upregulation of *MNX1* [11, 12].

This case highlights the complexities of classifying myeloid neoplasms with eosinophilia in the context of molecular abnormalities. While the patient's initial bone marrow showed only 6% blasts, molecular testing revealed an NPM1 mutation—an abnormality that, under the WHO 5th edition (2022) [7], is sufficient to establish a diagnosis of “AML with mutated NPM1,” regardless of blast percentage. In contrast, the WHO 2017 (4th edition) [13] requires a blast threshold of ≥ 20% to diagnose AML unless specific cytogenetic abnormalities (e.g., *t(8;21)*, *inv(16)*, and *t(15;17)*) are present. NPM1 mutations alone were not considered diagnostic under that system. The International Consensus Classification (ICC, 2022) [14] adopts a more permissive threshold of ≥ 10% blasts in the presence of recurrent genetic abnormalities, including NPM1. Thus, under both WHO 2017 and ICC criteria, this case would not meet criteria for AML and would more likely have been classified as a CEL or another eosinophilic myeloid neoplasm.

Although we acknowledge this classification discrepancy, the patient's disease course was clinically aggressive and more consistent with AML, including rapid deterioration and subsequent full-blown relapse. We, therefore, considered the WHO 5th edition framework to be the most appropriate basis for diagnosis in this case.

The fusion *ETV6*::*ACSL6* has previously been shown in some cases with AML, mostly secondary AML or relapsed AML [9]. Co-occurrence with an AML-defining genetic abnormality has been shown in two cases of AML with *DEK*::*NUP214*, where the *ETV6*::*ACSL6* fusion was present together with *DEK*::*NUP214* at relapse [8]. A previously reported case closely resembles ours, describing a patient who presented with hypereosinophilia, *t* (5; 12)(q31; p11.2), and 9% myeloblasts in the bone marrow, progressing to AML within months [14]. In that case, an *NPM1* mutation was identified at AML diagnosis and, retrospectively, also at the initial presentation of eosinophilia. Although the *ACSL6::ETV6* fusion transcript could not be detected by PCR, the presence of the fusion could not be definitively ruled out [14]. These cases indicate that *ETV6*::*ACSL6* and AML-defining abnormalities are not mutually exclusive, thus not qualifying for a specific disease entity. There is no evidence that *ACSL6*::*ETV6* could respond to imatinib treatment, unlike *PDGFR*-rearrangements, which is reasonable since there is no fusion protein with tyrosine-kinase effects.

In conclusion, acute leukemia must be considered in the differential diagnoses of severe eosinophilia, particularly when blast cells are present in the peripheral blood.

## Data Availability

The data used to support the findings of this study are available from the corresponding author upon reasonable request.

## References

[1] Akuthota P., Weller P. F. (2015). Spectrum of Eosinophilic End-Organ Manifestations. *Immunology and Allergy Clinics of North America*.

[2] Lampinen M., Carlson M., Håkansson L. D., Venge P. (2004). Cytokine-Regulated Accumulation of Eosinophils in Inflammatory Disease. *Allergy*.

[3] Groh M., Rohmer J., Etienne N. (2023). French Guidelines for the Etiological Workup of Eosinophilia and the Management of Hypereosinophilic Syndromes. *Orphanet Journal of Rare Diseases*.

[4] Larsen R. L., Savage N. M. (2019). How I Investigate Eosinophilia. *The International Journal of Literary Humanities*.

[5] Morales-Camacho R. M., Caballero-Velázquez T., Borrero J. J., Bernal R., Prats-Martín C. (2024). Hematological Neoplasms With Eosinophilia. *Cancers (Basel)*.

[6] Shomali W., Gotlib J. (2022). World Health Organization-Defined Eosinophilic Disorders: 2022 Update on Diagnosis, Risk Stratification, and Management. *American Journal of Hematology*.

[7] Khoury J. D., Solary E., Abla O. (2022). The 5th Edition of the World Health Organization Classification of Haematolymphoid Tumours: Myeloid and Histiocytic/Dendritic Neoplasms. *Leukemia*.

[8] Baldazzi C., Luatti S., Marzocchi G., Grassi A., Cavo M., Testoni N. (2022). t(5;12)(q31;p13)/ETV6::ACSL6 and t(6;9)(p23;q34)/DEK::NUP214 Concurrence in Acute Myeloid Leukemia: An Unusual Association of Two Rare Abnormalities. *Cancer Genetics*.

[9] Wu X., Cai H., Qiu Y., Li J., Zhou D. B., Cao X. X. (2020). ETV6-ACSL6 Fusion Gene in Myeloid Neoplasms: Clinical Spectrum, Current Practice, and Outcomes. *Orphanet Journal of Rare Diseases*.

[10] De Luca-Johnson J., Ninfea J. I., Pearson L. (2016). Myeloid Neoplasms With t(5;12) and ETV6-ACSL6 Gene Fusion, Potential Mimickers of Myeloid Neoplasm With PDGFRB Rearrangement: Case Report With Imatinib Therapy and Review of the Literature. *Case Rep Med*.

[11] Nilsson T., Waraky A., Östlund A. (2022). An Induced Pluripotent Stem Cell t(7;12)(q36;p13) Acute Myeloid Leukemia Model Shows High Expression of MNX1 and a Block in Differentiation of the Erythroid and Megakaryocytic Lineages. *International Journal of Cancer*.

[12] Waraky A., Östlund A., Nilsson T. (2024). Aberrant MNX1 Expression Associated With t(7;12)(q36;p13) Pediatric Acute Myeloid Leukemia Induces the Disease through Altering Histone Methylation. *Haematologica*.

[13] Swerdlow S. H. C. E., Harris N. L., Jaffe E. S., Pileri S. A. (2017). WHO Classification of Tumours of Haematopoietic and Lymphoid Tissues.

[14] Hofmans M., Delie A., Vandepoele K. (2018). A Case of Chronic Eosinophilic Leukemia With Secondary Transformation to Acute Myeloid Leukemia. *Leukemia Research Reports*.

